# Whole-Genome Sequence of *Pseudomonas xanthomarina* Strain UASWS0955, a Potential Biological Agent for Agricultural and Environmental Uses

**DOI:** 10.1128/genomeA.01136-16

**Published:** 2016-10-13

**Authors:** Julien Crovadore, Bastien Cochard, Gautier Calmin, Romain Chablais, Torsten Schulz, François Lefort

**Affiliations:** a Plants and Pathogens Group, Research Institute Land Nature and Environment, hepia, HES-SO University of Applied Sciences and Arts Western Switzerland, Jussy, Switzerland; b Faculty of Engineering and Architecture, HES-SO University of Applied Sciences and Arts Western Switzerland, Delémont, Switzerland; c Philotimo SA, Geneva, Switzerland

## Abstract

We report here the whole-genome shotgun sequence of the strain UASWS0955 of the species *Pseudomonas xanthomarina*, isolated from sewage sludge. This genome was obtained with an Illumina MiniSeq and is the second genome registered for this species, which is considered as a promising resource for agriculture and bioremediation of contaminated soils.

## GENOME ANNOUNCEMENT

*Pseudomonas xanthomarina* (1) is a Gram-negative, halotolerant, nonfluorescent species growing between 4°C and 40°C. This bacterium is aerobic, rod-shaped, and motile. Isolated from aquatic animals (1), plants (2, 3), rhizosphere (4), polluted soils (5, 6), oil-contaminated water (7), mine soil (8), or cold desert soil (9), it is able to degrade polycyclic aromatic hydrocarbons (5, 7) and insecticides (6) and oxidize arsenic (8). It also displays characteristics of plant growth–promoting rhizobacteria (PGPR), such as phosphate solubilization, indole-3-acetic acid production, siderophores production, and endophytism (2–4, 6). The strain UASWS0955 was isolated from the biomass of a sewage sludge treatment installation in Croatia. The identification by 16S rDNA sequencing pointed at *Pseudomonas xanthomarina* since this strain shared 99.9% identity with 15 *P. xanthomarina* strains in GenBank (10). Genomic DNA, extracted from a pure axenic culture according to an adapted protocol (11), was sheared in a 50-µL AFA microtube (Covaris, USA) in an S2 ultrasonicator (Covaris) to achieve an average fragment size of 350 bp. A library was created using the TruSeq DNA PCR-free library preparation kits (Illumina), and the insert size was checked in a Fragment analyzer (Advanced Analytical Technologies, Inc). Whole-genome shotgun sequencing was carried out within one Illumina MiniSeq run at 2 × 150-bp paired-end read length, using a MiniSeq mid-output kit. The sequencing yield reached 5,011,022 reads (756.66 Mb DNA), representing a genome coverage of 164-fold. After performing the quality control of the reads with FastQC (12), the genome assembly was computed with SPAdes genome assembler version 3.8.1 (13). The resulting contigs were arranged with BioEdit (14) and were analyzed by QUAST (15). The final assembly yielded 40 contigs (≥200 bp.), with a total genome length of 4,607,758 bp, a GC content of 62.96%, and an *N*_50_ value of 497,938 bp. PlasmidFinder (16) and plasmidSPAdes (17) did not find any plasmid. The gene annotation was carried out with the Prokaryotic Genome Annotation Pipeline (PGAP) (18) and RAST version 2.0 (19). RAST discerned 4,200 coding sequence (CDS) genes scattered in 459 subsystems, while PGAP identified 4,239 genes for 4,173 CDSs, 46 pseudogenes, and 66 RNA genes. No virulence, disease, or toxin genes were detected. The bacterium owns resistance genes against metals (arsenic, cadmium, chrome, cobalt, copper, mercury, zinc) and a few antibiotics (fosmidomycin, polymyxin, penicillin, fluoroquinolones). PGPR properties (20, 21) could be supported by four genes for plant auxin synthesis, 15 genes for PAPA antibiotic synthesis, a gene cluster for siderophore biosynthesis, a gene for an ACC-deaminase (lowering plant ethylene levels), 175 genes for chemotaxis and flagellar motility, and 40 genes for phosphorus metabolism, including phytase, inorganic pyrophosphatase, and inorganic phosphate transporter genes for phosphate solubilization. Various degradation pathways of aromatic compounds are provided by 56 genes that would allow growth in contaminated environments. Genes of amylase and cyanophycin synthetase could be of interest for industrial biotechnology. This genome, the second one for this species, will increase the knowledge on *Pseudomonas xanthomarina*.

### Accession number(s).

This whole-genome shotgun project was deposited at DDBJ/EMBL/GenBank under the accession number MDEM00000000. The version described in this paper is the first version, MDEM00000000.1. The 40 contigs have been deposited under the accession numbers MDEM01000001 to MDEM01000040. The DNA reads are available from the NCBI Sequence Reads Archives (SRA) under the accession number SRR4031069.
